# Depressive episode as initial symptoms of Perry syndrome

**DOI:** 10.1192/j.eurpsy.2023.1746

**Published:** 2023-07-19

**Authors:** A. Téllez Gómez, C. Pardo González, A. Sabater Ferragut, J. Ribes Cuenca

**Affiliations:** 1 Psychiatry and Clinical Psychology, Hospital Universitario y Politécnico La Fe, Valencia, Spain

## Abstract

**Introduction:**

Perry syndrome is a rare neurological disorder, characterized by atypical parkinsonian symptoms, sleep disturbances, central hypoventilation, weight loss, and psychiatric symptoms, especially apathy or depression. This syndrome is due to a TDP-43 proteinopathy as a result of a mutation in the DCTN-1 gene.

**Objectives:**

To present the case of a patient with a mutation in the DCTN1 gene, related to Perry syndrome, who debuted with several depressive episodes, with apathy and weight loss.

**Methods:**

A non-systematic literature review was conducted on PubMed database on depressive episodes related to Perry´s syndrome. The clinical case report was prepared through the review of the clinical record of the patient.

**Results:**

The authors present the case of a 49-year-old man, who contacted psychiatry for the first time 10 years earlier due to depressive symptoms, suffering multiple episodes. These episodes consisted of hypothymic mood, apathy, anhedonia and marked irritability, together with suicidal ideation, leading to several drug overdoses. He also presented disruptive behaviors, such as abusive drinking and aggressiveness. These episodes responded to antidepressants at medium doses, although maintaining several relapses. Given this, it was decided to introduce valproic acid as a mood stabilizer, with good tolerance.

In parallel, the patient’s mother, who had also suffered from depressive episodes, began with dementia symptoms, after which it was decided to request a genetic study. In this context, a mutation, similar in both patients, was observed in the DCTN1 gene, related to Perry syndrome.

In the case of the patient presented, no other associated alterations were found, neither in the neurological examination or in the rest of the tests performed (polysomnography without notable alterations, functional imaging tests without pathological findings).

**Conclusions:**

Neurological diseases as Perry syndrome can show depressive symptoms and other behavioral changes at the beginning, developing the rest of the symptoms (parkinsonism, weight loss or central hypoventilation) several years after the onset of the symptoms. It must be taken into account in patients with a family history of mutations or atypical depressive symptoms. It should also be assessed in terms of genetic counseling.

**Disclosure of Interest:**

None Declared

